# Oldest *Varroa* tolerant honey bee population provides insight into the origins of the global decline of honey bees

**DOI:** 10.1038/srep45953

**Published:** 2017-04-10

**Authors:** L. E. Brettell, S. J. Martin

**Affiliations:** 1 School of Environment and Life Sciences, The University of Salford, Manchester, M5 4WT, UK

## Abstract

The ecto-parasitic mite *Varroa destructor* has transformed the previously inconsequential Deformed Wing Virus (DWV) into the most important honey bee viral pathogen responsible for the death of millions of colonies worldwide. Naturally, DWV persists as a low level covert infection transmitted between nest-mates. It has long been speculated that *Varroa* via immunosuppression of the bees, activate a covert infection into an overt one. Here we show that despite *Varroa* feeding on a population of 20–40 colonies for over 30 years on the remote island of Fernando de Noronha, Brazil no such activation has occurred and DWV loads have remained at borderline levels of detection. This supports the alternative theory that for a new vector borne viral transmission cycle to start, an outbreak of an overt infection must first occur within the host. Therefore, we predict that this honey bee population is a ticking time-bomb, protected by its isolated position and small population size. This unique association between mite and bee persists due to the evolution of low *Varroa* reproduction rates. So the population is not adapted to tolerate *Varroa* and DWV, rather the viral quasispecies has simply not yet evolved the necessary mutations to produce a virulent variant.

The ecto-parasitic *Varroa destructor* mite in combination with its associated viruses, most notably Deformed Wing Virus (DWV) has been associated with the death of millions of European honey bee colonies (*Apis mellifera*) across the world^1^,^2^. The *Varroa* mite, initially a pest of the Asian honey bee (*Apis cerana*) jumped the species barrier in the 1950s and subsequently spread around the world facilitated by modern beekeeping practices^3^,^4^, where it has changed the viral landscape in honey bee populations by transforming DWV from a relatively harmless pathogen present in low prevalence and titre to a deadly virus now present in large amounts in virtually every *Varroa* infested colony in the world^1^,^5^,^6^.

In the absence of DWV, a very large mite population are required to kill a mature honey bee colony^7^ due to haemolymph removal during feeding. Since healthy colonies in South Africa have been reported to regularly maintain mite populations of 30,000–50,000^8^. Whereas, in the presence of DWV the number of mites needed to kill a mature colony becomes vastly reduced to just a couple of thousand mites^6^,^9^,^10^. This is due to the premature death of both developing honey bee brood^11^ and adult bees^12^ that become infected with DWV via the feeding activities of *Varroa*^11^. This causes the colony to enter a downward spiral of bee losses, ultimately resulting in the collapse of the colony. However, colony losses only start to occur several years after the mites’ arrival. During this period both DWV prevalence and load increase accompanied with a loss of viral diversity^5^,^13^. However, the mite’s initial role in DWV transmission and amplification in the bee remains conjecture. The two main theories are; the mite’s feeding activity activates covert virus within the host^14^,^15^,^16^,^17^ or the mite has to encounter and feed on a bee (adult or brood) which is suffering from an overt infection. This allows the feeding mite to become infected with sufficient DWV particles (including virulent variants) that allows the infection to be passed to another bee, thus establishing a new viral transmission route^1^. These two theories still persist as it is exceedingly difficult for studies to separate out the effects of the bee, mite or virus, since bees or mites cannot be easily reared in large numbers on artificial media to ensure they are guaranteed 100% viral free.

It is now well established that *Varroa* has transformed DWV from an inconsequential virus to the most important honey bee viral pathogen but the nature of the interaction is still the subject of much contention. Under laboratory conditions the feeding activity of *Varroa* has been shown to increase the viral load in the developing bee^18^, which numerous studies have attributed to a *Varroa*-induced down-regulation of host immune gene transcription^16^,^15^. Yang and Cox-Foster^17^ suggested this is a response to bacterial infections introduced at the mites’ feeding site, however Nazzi *et al*.^18^, put it down to DWV replication itself. Conversely other studies found honey bee viral infections to have little or no effect^19^,^20^, or even cause an up-regulation of immune genes^21^. Part of this confusion may lie in the fact that many studies focus on immune genes known to be active against bacterial infections, rather than antiviral immunity, which is more complex (e.g. involving virus-derived small interfering RNAs and piwi-interacting RNAs) and currently is poorly understood^22^,^23^.

Prior to the global spread of *Varroa* DWV was associated with death in a very small number of honey bee colonies. The virus was detected in dead adult bees from Britain, South Africa^24^ and Belize (Brenda Ball personal communication), but these events were very rare considering the large numbers of dead bees sent to Rothamsted Research centre for viral testing by Bailey & Ball between the 1960s and 1990s. In each case where DWV was detected the viral loads were very high (>10^8^ particles/bee) i.e. an overt infection, as this is the detection limit of the ELISA methods used to detect honey bee viral pathogens. Furthermore, a study in Hawaii of 341 colonies discovered one *Varroa* free colony that had overt DWV titres^5^ the following year this remote colony was dead. Therefore, DWV can emerge as an overt infection in the absence of *Varroa*, but this is a rare event.

The initial aim of the study was to discover how the oldest *Varroa* tolerant European honey bee (*Apis mellifera ligustica*) population in the world has survived with *Varroa* for the past 32 years. This bee population was established in 1984 on the remote Brazilian island of ‘Fernando de Noronha’^25^ where the 20–40 managed colonies have never been treated for *Varroa* and no unexpected colony deaths have ever been reported. This honey bee population has maintained a consistently high *Varroa* infestation level^25^,^26^ with seemingly no effect on the honey bees. This provides a perfect opportunity to study mite tolerance mechanisms and impact of DWV, if present. So specifically, we investigated the presence or influence of DWV and how the stable *Varroa* population is maintained.

## Results

### DWV analysis

**Figure 1:**
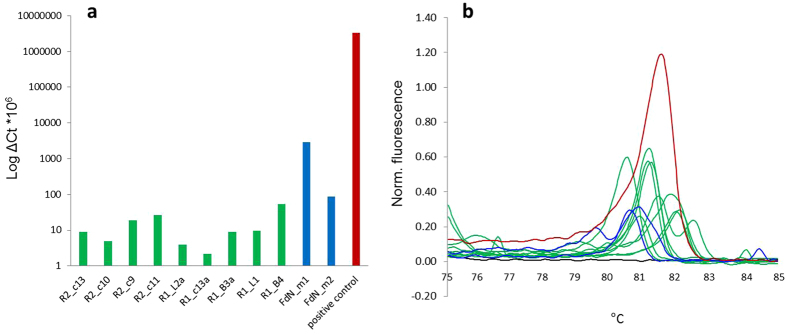
RT-PCR HRM results of all DWV positive samples using primers developed by Highfield *et al*.^46^ with a known DWV (type A) positive asymptomatic honey bee sample (red) and a no template control (black). (**a**) DWV levels in each colony relative to an actin control shown as ΔCt *10^6^ values to enable visualisation of low level samples (**b**) HRM profiles generated indicate diverse DWV genotypes are present in the population.

Surprisingly DWV was detected in honey bees on Fernando de Noronha using both highly sensitive High resolution melt (HRM) analysis, Agarose Gel Electrophoresis and Sanger sequencing of purified gene fragments in five out of twelve colonies sampled in July 2015, and four out of six sampled in May 2016. qRT-PCR of actin gene fragments showed all samples to contain intact RNA (C_t_ = 18.15, S.D. = 2.45) (Supplementary Table S1). DWV loads were at the borderline of detection limits (Fig. 1a, Supplementary Table S1), far below the level where accurate quantification was possible and considerably lower than the positive control, which was a DWV positive, newly emerged bee which had never been parasitised by *Varroa,* which is considerably lower than what occurs in a mite parasitised bee. Replicate samples did not always provide consistent results due to the very low DWV loads present i.e. at the limits of detection. Therefore, the specific numbers of DWV infected colonies should not be given much weight. The variation in HRM profiles produced using all bee and mite DWV positive samples (Fig. 1b) indicate that the DWV sequences present are variable and no one dominant variant exists in this population, a scenario typical of *Varroa* free honey bee populations^5^,^13^. During the sampling and reproductive studies which involved opening over 75% of all managed hives on the island no bees with deformed wings were observed.

**Figure 2:**
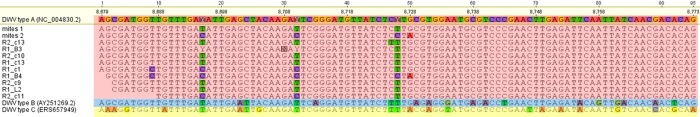
Geneious alignment of a 95 bp fragment amplified from all positive honey bee and mite samples aligned to DWV type A (pink). DWV types B (blue) and C (yellow) are also shown.

The resulting fragments from HRM analysis were subjected to Sanger sequencing which confirmed that DWV had been amplified from all positive samples (Fig. 2). Variation was seen between samples but the dominant variant found in both bees and mites was closest to the type A variant (Fig. 2).

### Honey bee and *Varroa* populations

**Figure 3:**
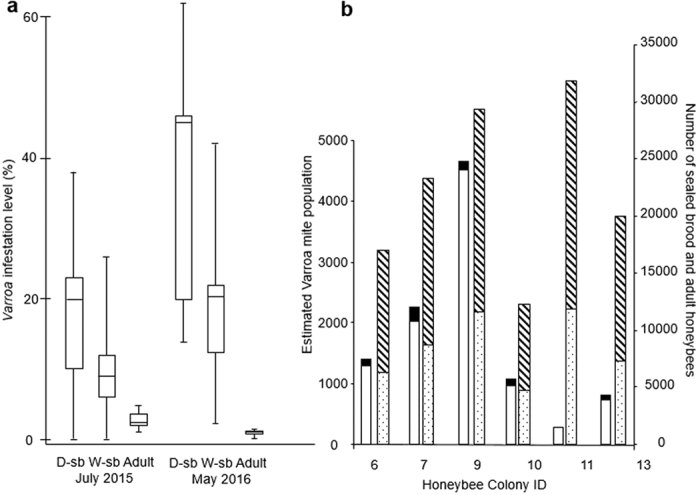
(**a**) *Varroa* Infestation levels of drone (D-sb) sealed brood, worker (W-sb) sealed brood and adult workers sampled at July 2015 & May 2016 and (**b**) the calculated mite population in sealed brood (clear bar) and on adult bees (black bar) alongside the number of sealed brood (dotted bar) and adult bees (striped bar) in each of the six colonies studied in 2016.

A total of 276 drone and 921 worker sealed brood cells from six colonies were opened. Of these, 106 drone and 201 worker sealed brood cells were infested with one or more *Varroa* mites. The infestation levels of sealed brood and adult workers were variable; both between colonies and month of collection (Fig. 3a) as previously found^26^. All colonies were infested, with adult bee infestation levels much lower (1–2%) than found in the worker (10–20%) or drone (23–38%) brood cells. In May 2016, the six study colonies contained an average of 8400 (±2865 SD; range 4684–11839) sealed brood cells, 13894 (±4560 SD; range 7655–19982) adult bees and 1749 (±1565 SD; range 290–4647) mites per colony (Fig. 3b).

### *Varroa* reproduction

The developmental times of the mite offspring based on the age of bee pupa are all indistinguishable to that found in previous studies^27^,^28^ (Supplementary Fig. 1). Furthermore, the average number of eggs laid in worker cells (4.9) and drone cells (5.3) is typical for *V. destructor*^29^. However, the highest non-reproduction rates and high offspring mortality rates combined to produce the lowest number of viable female offspring produced per mite (0.54 in worker & 1.6 in drone sealed brood) ever recorded (Supplementary Table S2). These numbers fell to 0.39 and 1.0 in worker and drone cells respectively that were invaded by two of more mother mites (Supplementary Table S2).

## Discussion

The viral analysis indicates that DWV is present among the ‘Fernando de Noronha’ honey bee and mite population, but the levels are very low and genotype (strain) diversity is high. Sequencing of HRM products detected predominantly DWV type A despite the HRM analysis of the same samples often indicating multiple variants. This apparent paradox arises due to the much higher sensitivity of HRM compared to sequencing of samples containing very low amounts of virus. We know from previous experience, that even using high-depth NGS methods it is very difficult to detect multiple variants that we know to be present using HRM analysis in honey bee samples from *Varroa* free areas with low level DWV infections. It should also be noted that although the sequenced RdRp region is closest to the type A master variant, the dominant DWV genomes could be recombinants and contain structural genes more similar to types B or C. This pattern of DWV, i.e. low amounts and diverse genotypes, has also been seen in *Varroa* free honey bee populations on Colansay Island, Scotland^13^ and Hawaii, USA^5^. However, this is the first honey bee population where DWV is associated with a *Varroa* infested honey bee population for a long period of time (32 years) and no virulent strain has appeared. This confirms for the first time that the feeding activity of *Varroa* cannot activate DWV replication, either by immunosuppression or by any other mechanism such as the injection of proteins during mite feeding^14^. This population has lived with *Varroa* since its establishment in 1984^25^, and so must have also acquired the original DWV infection (containing no virulent variants) at that time, either through the imported queens, the Africanized workers originally used to initiate the colonies or the *Varroa* mites which arrived with them, as no bees have been moved on to the island since 1984. If *Varroa*-induced viral replication were taking place viral loads would be much higher and killing the bees.

We propose the explanation for this population’s survival may be mere probability. The mechanism underlying the *Varroa* induced transformation of DWV infection in bees from a genetically variable and low titre inconsequential virus to a deadly virus dominated by a virulent genotype has until now been unknown. The data from this study, along with data from Ryabov *et al*.^13^, which showed that the decrease in viral variation occurred in the bee rather than the mite, suggests that in order for a virulent variant to become established it must first become an overt infection within a bee (pupae or adult). This then allows *Varroa* to then transmit sufficient amounts of this virulent variant throughout the population. Although RNA viruses exhibit high mutation rates and no proof reading activity leading to extremely fast evolution of their quasispecies^30^ amplification of a lethal DWV variant, which goes on to kill a colony is a rare occurrence. Under natural conditions, without *Varroa* to spread the virus, the overt infection would likely go unnoticed as the colony would quickly die without spreading the virus further. Pre-*Varroa*, isolated colony deaths associated with overt infections of DWV were reported in Hawaii^5^, UK and South Africa^24^ and Belize (Brenda Ball, personal communication). It has now been shown^13^ that the appearance of a virulent DWV-variant occurs within the bee, not the mite, which appears to be acting only as a mechanical vector. However, the conditions required for the sudden amplification of the type A variant within the bee remains unknown. Although once present in the honey bee population it is vectored very efficiently by the mites.

So the honey bee population in Fernando de Noronha has thus far evaded the catastrophic consequences of DWV and *Varroa* because the incredibly small and isolated population size (ca. 20–40 colonies) has meant that there hasn’t yet been sufficient time for a virulent variant to have become established in a colony. The estimated mite populations in the colonies would no-doubt result in the rapid death of the colonies if a virulent genotype of DWV was to emerge, since up to 42% of the worker brood can be infested by *Varroa*, levels never observed in healthy hives of European honey bees. Moreover it is just a matter of time before an overt outbreak of a virulent variant appears that has the capability to spell disaster for the bees of Fernando de Noronha. It also explains why when in 1997 six queens were transferred from Fernando de Noronha to Germany to head colonies and study whether heritable hygienic behaviour is responsible for their *Varroa* tolerance^31^. Although no difference in hygienic abilities compared to the local population were found indicating no genetic basis for the tolerance is present. These colonies all died during the winter or early spring (Peter Rosenkranz, personal communication) since the bees and mites would for the first time be exposed to the virulent DWV strains^5^,^32^ circulating in the local bee population.

The second insight from this study is the co-evolution of the honey bee and *Varroa* mites on Fernando de Noronha, free of any influence from DWV. Again this is a globally unique situation. Over the past 25 years [25, 26, this study] the initial high infestation rates on adult bees has fallen from 25% to just a few percent (this study). However, the average infestation rates of the worker (18%-1996, 20%-2012 & 20%-2016) and drone (38%-1996, 45%-2012 & the 38%-2016) sealed brood has remained remarkably high and stable. It is estimated that currently mite populations range from 290 to 4684 per colony, which for some colonies is well above the economic threshold of 1000–2000. It was originally suggested that the Japanese haplotype of *Varroa* was less ‘virulent’ than the Korean haplotype^33^. This was proposed as the reason for *Varroa* tolerance among the Fernando de Noronha population^34^,^35^ and in Africanized bees^36^. However, the Korean haplotype is now found in Africanized bees without any loss of tolerance^37^. This study found that the number of eggs laid and developmental timing in both worker (and drone brood) are indistinguishable from those of the Korean haplotype. However, adult mite and offspring mortality are higher than reported in other studies (Supplemental Table S2). This results in only 0.54 viable i.e. mated, female offspring being produced per reproductive cycle, which is one of the lowest values ever recorded. As the total number of reproductive cycles is around 2 to 3^38^,^39^ the mite population is unable to significantly increase within worker cells relying on the more limited drone cells. Furthermore, both in this study and all previous studies a large drop in reproductive success occurs when increasing numbers of mothers invade a cell (Supplemental Table S2, Supplemental Fig. 2^26^), which can potentially led to a stable mite population when mite reproductive success in worker brood is low^40^, as found in this population.

Currently known *Varroa* tolerant populations are surviving due to increased swarming^41^,^42^ or superinfection exclusion^43^. However, on Fernando de Noronha the mite and bee populations are both able to persist without any severe effects, however, the dark spectra of DWV lurks in the background, ready to decimate this small unique island population.

## Methods

### Mite & honey bee samples

The island of Fernando de Noronha in North-eastern Brazil (S 3° 50′ 47.5″; W 32°25′ 40.8″), lies 350 km from the mainland and has a tropical climate. All honey bee colonies are maintained in Langstroth hives in various combinations of one or two brood boxes and one or two supers as their size dictates. No queen excluders are used and colonies are allowed to swarm and re-queen themselves naturally. During the past 32 years the island population has been confirmed using mitochondrial DNA and isozyme analysis^25^ as belonging to *Apis mellifera ligustica*. This is further supported by their very mild temperament and yellow colour.

During the first visit (1–7 July, 2015), 13 of the 20 managed colonies across the three apiaries on island were sampled. From each colony 40–100 adult bees were collected from the brood comb of each colony. These were used to roughly estimate the *Varroa* infestation level and determine the presence or absence of DWV (see below). In addition, from 10 colonies approximately 25 drone (if present) and 50 worker sealed brood cells were opened to determine the proportion of *Varroa* infested cells. The aim of the second visit (18–23 May, 2016) was to obtain further samples for DWV analysis and detailed data on *Varroa*’s ability to reproduce in both drone and worker cells. Therefore, six colonies in the apiary maintained by Lidia Albuquerque were chosen at random. For each, a photograph of every brood frame was taken to allow an estimation of colony size using methods from Calis *et al*.^44^ and Martin^45^. Approximately 100 bees from a brood frame were collected for viral analysis and determining the proportion of phoretic mites. Lastly a single frame that contained sealed worker brood older than 7 days, where possible, was removed for the *Varroa* reproductive study.

### Detection of DWV

All adult honey bees were killed by freezing, and transport to the laboratory using a Dry Vapour Shipper that maintains the samples at −186 °C, prior to long term storage at −80 °C.

Pools of 20 adult honey bees were taken per colony and searched for mites. Where found these were removed and stored separately. Pooled bees and two pools of mites, one removed from sealed brood and the other removed from adult hive bees were crushed to a fine homogenous powder in liquid Nitrogen using a sterile pestle and mortar. 30 mg of powder was used for RNA extraction. Remaining bee powder was stored at −80 °C. Total RNA was extracted using the RNeasy mini kit (Qiagen) following manufacturers’ instructions eluting in 30 μl RNase free water followed by quantitation using a Nanodrop 8000 (Thermo Scientific). Samples were then standardised to 50 ng/μl. cDNA was synthesised from 200 ng RNA using the Quantitect Reverse Transcription kit (Qiagen) following manufacturers’ conditions. The resulting cDNA was then used for PCR and HRM analysis which was performed using the SensiFAST HRM kit (Bioline). Reactions contained 1× HRM mix, 7.5 pmol each primer (DWVQ R1 and F1^46^) and 4 ul cDNA in a final volume of 20 μl. Reactions were run on a Rotor-gene Q Thermocycler (Qiagen) using an initial denaturation of 95 °C for ten min, followed by 40 cycles of 95 °C for 15 sec, 54 °C for 10 sec and 72 °C for ten sec. A HRM curve was produced by a final dissociation step rising from 65 °C to 95 °C with 0.1 °C increments acquiring to the HRM channel. HRM PCR products were run on a 2% agarose gel stained with 0.001% GelRed to confirm the correct sized band had been amplified. Fragments were excised from the gels using a sterile scalpel and PCR products cleaned up using the Zymoclean gel DNA recovery kit (Zymo) followed by Sanger sequencing using the DWVQ forward primer by Source Bioscience (Rochdale). Electropherograms were inspected using FinchTV and converted to FASTA files which were aligned to DWV types A (NC_004830.1), B (AY251269.2) and C (ERS657949) using Geneious v8.1.2 (Biomatters). qRT-PCR reactions were also carried out to amplify the actin control gene in all samples using the SensiFAST SYBR No Rox One-step kit (Bioline). Reactions contained 50 ng RNA which had been DNase treated using a RQ1 Dnase 1 kit (Promega), 7.5 pmol each primer (actin f1 and r1^46^), 1x SYBR onestep sensimix, 5 units of RNase inhibitor and 0.2 μl Reverse transcriptase. DWV loads calculated from RT-PCR and HRM data relative to actin expression (qRT-PCR) were then calculated as ΔCts and compared to a known DWV positive newly emerged bee which had developed free of *Varroa*.

### *Varroa* reproduction measurements

Each frame had all sealed drones cells (if present) and between 128–236 worker cells aged Pink-eyed or older (140 hours post-capping) carefully opened under a x5 binocular microscope using watch-maker forceps. If a cell was infested by *Varroa* the entire cell contents were removed onto a microscope slide using a fine ‘wetted’ paint brush. The development stage of each bee and any mites present were determined using the ontogenic development charts^27^,^47^,^48^. In addition, the female deutonymphs were classified into 4 stages (small & medium mobile; large immobile; moulting) using Figure 11 in Dietemann *et al*.^47^ as a guide. Moulted skins of the male and females were used to confirm the presence of a new adult male or female within the cell. The moulted skins and development age of each mite offspring allows the reconstruction of each mite family so mortality rates and other reproductive behaviours can be determined^27^,^48^. Cells containing a single mite family were analysed separately from all cells invaded by two or more mites. Several key reproductive factors, egg number, development times, mortality rates and number of viable adult female offspring produced are calculated and compared with previous studies conducted in European^27^,^49^,^50^ and Africanised honey bees^51^,^52^,^53^,^54^ that critically used the same methodology.

## Additional Information

**How to cite this article:** Brettell, L. E. and Martin, S. J. Oldest *Varroa* tolerant honey bee population provides insight into the origins of the global decline of honey bees. *Sci. Rep.* **7**, 45953; doi: 10.1038/srep45953 (2017).

**Publisher's note:** Springer Nature remains neutral with regard to jurisdictional claims in published maps and institutional affiliations.

## Supplementary Material

Supplementary Information
