# Supplementary material for: Oldest Varroa tolerant honey bee population provides insight into the origins of the global decline of honey bees
Source: Sci Rep. 2017 Apr 10;7:45953. doi: 10.1038/srep45953 (PMC5385554; doi:10.1038/srep45953)
Supplement: Supplementary Information [file srep45953-s1.pdf]

Oldest *Varroa* tolerant honey bee population provides insight into the origins of the global decline of honey bees

L E Brettell* and S J Martin

School of Environment and Life Sciences,

The University of Salford, Manchester M5 4WT, UK

*corresponding author

Supplementary Information

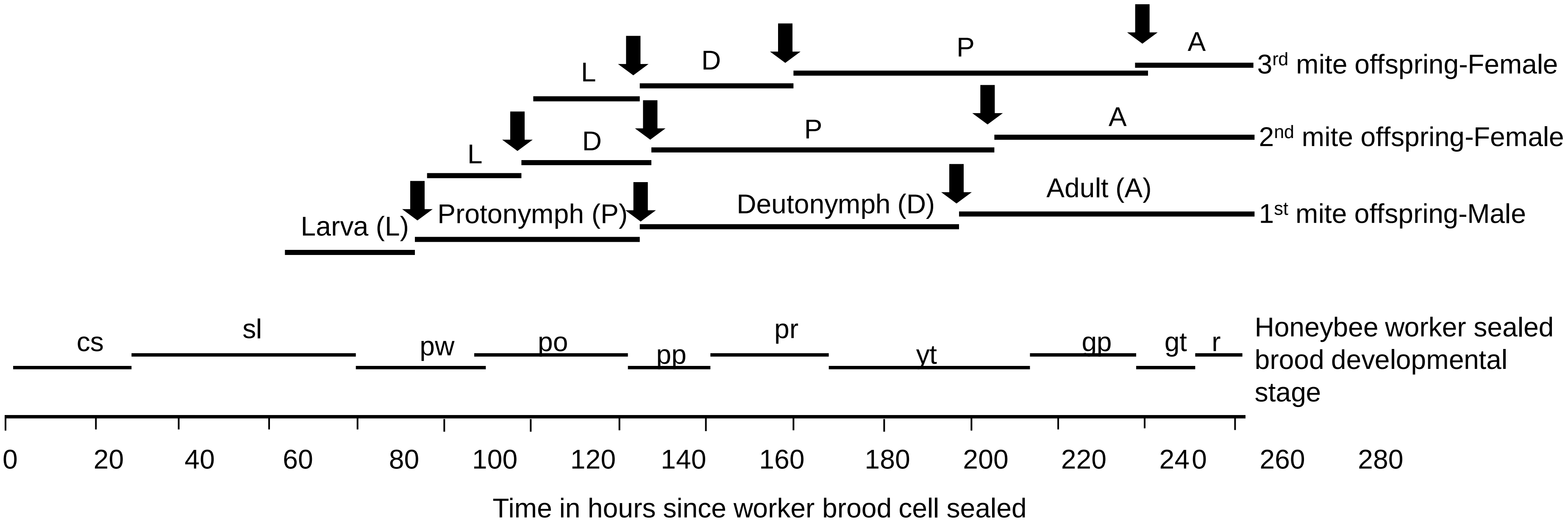

Supplementary Figure 1. The development and timing of Varroa in relation to the development and timing of the honey bee inside the sealed brood cell adapted from Martin [27]. Honey bee development stages: cs=cocoon spinning, sl= stretched larva, pw=pupa with white eyes, po=pale eyes, pp=pink eyes, pr=purple eyes, yt=yellow thorax, gp=grey wing pads, gt=grey thorax, r=resting adult.

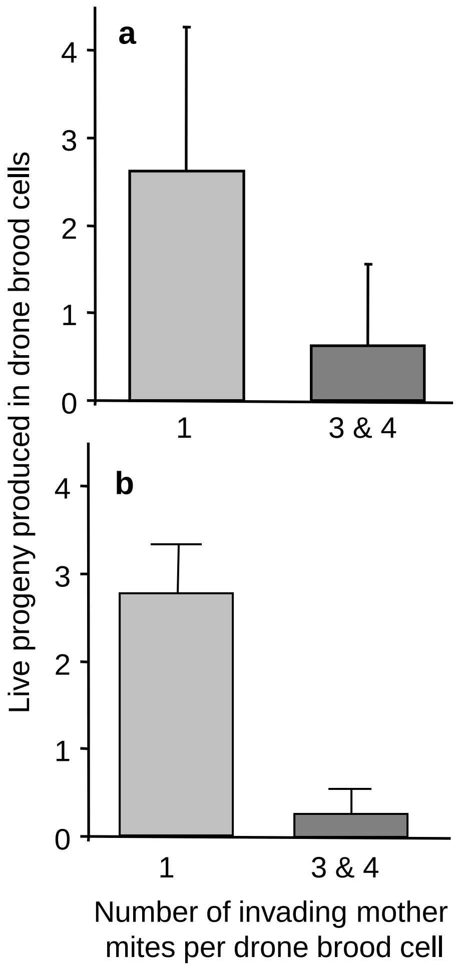

Supplementary Figure 2. Comparison of the number of live progeny produced in drone brood per invading mother in a) this study and b) a previous study [26], calculated using the method used in [26]. This indicates the importance of using the same method when comparing mite reproductive data across studies.

collection	sample	Ct (DWV)	Ct (actin)
Jul-15	R1_c10	neg	19.48
Jul-15	R1_D1	neg	13.71
Jul-15	R1_B1	neg	18.12
Jul-15	R1_B2	neg	16.82
Jul-15	R1_L2	34.43	16.49
Jul-15	R1_c13	35.24	16.43
Jul-15	R1_B3	34.11	17.34
Jul-15	R1_c1	33.29	16.61
Jul-15	R1_B4	31.3	17.11
Jul-15	R1_c9	neg	15.35
Jul-15	R1_B5	neg	18.01
May-16	R2_c6	neg	17.87
May-16	R2_C7	neg	18.48
May-16	R2_c13	34.42	17.67
May-16	R2_c10	35.76	18.12
May-16	R2_c9	34.12	18.44
May-16	R2_c11	34.4	19.22
May-16	*Varroa* 1	33.83	25.38
May-16	*Varroa* 2	36.55	23.04
	positive control	17.59	19.32

Supplementary Table S1. Presents the mean DWV and actin Ct values for each colony tested as well as the two pooled *Varroa* samples and a positive control of an asymptomatic newly emerged worker bee which had been parasitised by *Varroa* during development.

	Worker sealed brood				Drone sealed brood			
**Category***	This study	EHB †	African [53]	AHB [52]	This study	EHB †	African [53]	*A.cerana* [54]
**Dead Mothers (all)**	11%	2	6	2	18%	5	6	
**Non-reproductive >po**	19%	10	13	12	10%	3	2	0-2
**Viable mothers >po**	70%				65%			
**Only males >po**	6%	9	15	11	20%	14	20	0-2
**Viable offspring (>gp) Live male + female**	40%	63	51	43	53%	63	59	94-99
**Mean # of eggs laid**	4.9	4.9	4.5	4.38	5.3	5.5	4.9	
**No of viable females per mother >gp**	0.54	0.9	0.9	0.6-0.7	1.6	1.9-2.1	2.2	4.6
**No of viable females from multiple infested cells**	0.39	1.0			1.0	1.6		

Supplementary Table S2. Presents the various reproductive categories for the J type mites in this study compared to other studies using a similar methodology. po=pale eyed pupa and gp=grey wing pads, referring to host honey bee life stages. *Percentages will not add up to 100 since they are considering different sub-sets of data for each calculation. †=recalculated from [27,49].
